# Verbascoside down-regulates some pro-inflammatory signal transduction pathways by increasing the activity of tyrosine phosphatase SHP-1 in the U937 cell line

**DOI:** 10.1111/jcmm.12524

**Published:** 2015-03-21

**Authors:** Mirko Pesce, Sara Franceschelli, Alessio Ferrone, Maria Anna De Lutiis, Antonia Patruno, Alfredo Grilli, Mario Felaco, Lorenza Speranza

**Affiliations:** a Department of Psychological, Humanistic and Territorial Sciences, University G. D’AnnunzioChieti, Italy; b Department of Medicine and Science of Aging, University G. D’AnnunzioChieti, Italy

**Keywords:** verbascoside, Tak-1, SHP-1, iNOS, COX 2

## Abstract

Polyphenols are the major components of many traditional herbal remedies, which exhibit several beneficial effects including anti-inflammation and antioxidant properties. Src homology region 2 domain-containing phosphatase-1 (SHP-1) is a redox sensitive protein tyrosine phosphatase that negatively influences downstream signalling molecules, such as mitogen-activated protein kinases, thereby inhibiting inflammatory signalling induced by lipopolysaccharide (LPS). Because a role of transforming growth factor β-activated kinase-1 (TAK1) in the upstream regulation of JNK molecule has been well demonstrated, we conjectured that SHP-1 could mediate the anti-inflammatory effect of verbascoside through the regulation of TAK-1/JNK/AP-1 signalling in the U937 cell line. Our results demonstrate that verbascoside increased the phosphorylation of SHP-1, by attenuating the activation of TAK-1/JNK/AP-1 signalling. This leads to a reduction in the expression and activity of both COX and NOS. Moreover, SHP-1 depletion deletes verbascoside inhibitory effects on pro-inflammatory molecules induced by LPS. Our data confirm that SHP-1 plays a critical role in restoring the physiological mechanisms of inducible proteins such as COX2 and iNOS, and that the down-regulation of TAK-1/JNK/AP-1 signalling by targeting SHP-1 should be considered as a new therapeutic strategy for the treatment of inflammatory diseases.

## Introduction

The widespread trend towards the use of substances of natural origin and the growing rejection of synthetic substances by consumers, together with more awareness and the purposeful use of antibiotics and chemotherapeutic agents, has focused interest on the scientific application of herbs and their extracts 1. These products are able to enhance body’s defence endurance against chemical, biological and environmental factors through different routes. These ‘natural substances’ are low molecular weight organic compounds found in plants that activate a defence system against physiological and/or environmental stress 2–4. Such compounds have antioxidant, antimicrobial and immunomodulatory properties and derive from secondary metabolites which belong mainly to the category of terpenes, terpenoids, alkaloids, phenols and flavonoids 5–7. In particular, polyphenols compound are secondary metabolites widely distributed in the plant kingdom, with many biological properties, anti-allergic, anti-atherogenic, anti-inflammatory, antimicrobial and antioxidant 8. Of particular interest in recent years are the phenylpropanoid glycosides (PPGs, also synonymous with phenylethanoid glycosides) which are water soluble derivatives of phenylpropanoids (PPs), a large group of natural polyphenols 9. Verbascoside, a polyphenol present in many plants used for food, flavouring and medicines like olive and mullein, shows the highest scavenger activity among PPG tested 10,11, as well as a high antioxidant power, even in comparison with other natural phenolic compounds 12. PPGs are a class of more than 200 plant-derived polyphenols acting as potent antioxidants by direct scavenging of reactive oxygen species (ROS) and reactive nitrogen species 13. Interest in the biological properties of protein tyrosine phosphatases (PTPs), which are encoded by more than 100 genes in the human genome, continue to grow at an increasing rate. In particular, two cytoplasmic tyrosine phosphatases composed of two Src homology 2 (SH2) NH2-terminal domains and a C-terminal protein-tyrosine phosphatase domain, referred to as SHP-1 and SHP-2 are known to modulate different cellular functions. More recently, a role has been suggested for SHP-1 and SHP-2 in controlling cell survival by oxidative stress pathway regulation 14. PTPs belongs to a family of molecules that catalyses the removal of phosphate groups from phosphotyrosine residues on proteins 15. The perturbation of PTP signalling can lead to abnormal accumulations of tyrosine-phosphorylated proteins, which can disrupt normal cell proliferation and differentiation. SHP-1 (PTPN6, SHPTP1, HCP or PTP1C) is a cytosolic phosohatase that is primarily expressed in hematopoietic and epithelial cells. SHP -1 is a negative regulator in various signalling pathways and acts as a negative regulator of cytokine signalling *via* STAT1, STAT3, and STAT6 16. Transforming growth factor β-activated kinase-1 (TAK-1), a well-characterized mitogen-activated protein kinase (MAPK) kinase family member, has emerged as a key regulator of signal transduction cascades leading to the activation of the transcription factors NF-κB and AP-1 17. Multiple lines of evidence support the pro-inflammatory molecule known as bacterial lipopolysaccharide (LPS), activates multiple protein kinases such as p38, JNK, IKK-b, and PKB/Akt *via* TAK1 18,19. In several studies, the activation of transcription factor AP-1 in response to MAPK phosphorylation has also been reported. These intracellular signalling pathways that control the expression of the genes involved in inflammatory responses might act as therapeutic targets in a wide range of inflammatory diseases 20. In this study, we used U937 cells to determine the molecular mechanisms underlying the inhibitory effect of verbascoside on the LPS-induced inflammatory response. We examined the effect of verbascoside on the activation of SHP-1 protein and AP-1 signalling. In addition, because a role by TAK1 in the upstream regulation of JNK and AP-1 signalling molecule has been well demonstrated, we investigated the effect of verbascoside on these proteins upon LPS stimulation. Our findings demonstrate that verbascoside can exert an anti-inflammatory effect on U937 cells by acting on SHP-1/TAK1 signalling.

## Materials and methods

### Cell culture

U937 mononuclear cells were purchased from American Type Culture Collection (Manassas, VA, USA). The cells were cultured in a 5% CO_2_ atmosphere in RPMI 1640 medium (Gibco, Invitrogen) containing 10% foetal calf serum, 100 ng/ml streptomycin, 100 U/ml penicillin and 2 mM L-glutamine. Cells derived from the same freeze-down batch were thawed, grown and seeded (at 2 × 10^5^ cells per well) onto six-well tissue culture plates and cultured in medium with and without 50 μM Verbascoside, and LPS (10 μg/ml). Pure verbascoside was dissolved in PBS, and the stock solution was stored at −80°C prior to use. To obtain the desired final concentration, the stock solution was diluted in cell culture medium.

### Cell viability assay

NAD(P)H-dependent oxidoreductase enzymes in viable cells convert MTT into the purple insoluble formazan which has an absorbance maximum near 570 nm. Thus, formazan formation is directly proportional to the amount of viable cells. U937 cells were seeded on 96-well plates at a density of 8 × 10^3^ cells/well, cultured and treated according to the method described above 21. MTT reduction was measured on an ELISA reader (Bio-Rad, Hercules, CA, USA). Values are expressed as a percentage of the control value.

### Silencing

U937 cells (4 × 10^5^) were transiently transfected with SMART pool PTPN6 (L-009778-00-0005) (GenBank accession number NM_002228), Non-Target (D-001810-10-05) or Gadph siRNA (D-001830-01-20) (Dharmacon, Inc., Chicago, IL, USA), each at a final concentration of 25 nM, using DharmaFECT II transfection reagent (Dharmacon, Inc.) by following the manufacturer’s instructions. Cells were incubated in the transfection medium for 24 hrs. Afterwards the cells were pre-treated with verbascoside 50 μM and stimulated with LPS 10 μg/ml and incubate for another 24 hrs (for mRNA analysis) and 48 hrs (for protein analysis).

### Quantitative Real-time PCR

A quantitative Real-time PCR assay was carried out in an Eppendorf Mastercycler EP Realplex (Eppendorf AG) as described previously 22. Briefly, preliminary PCR were run to optimize the concentration and ratio of each primer set. For all the cDNA concentration templates 2 μl was used in a 20 μl Real-time PCR amplification system of SYBR Green Real Master Mix Kit according to the manufacturer’s directions. Primers for human PTPN6, iNOS, COX2 and GAPDH as the control were designed using GeneWorks software (IntelliGenetix, Inc., Mountain View, CA, USA). The primer pairs used were as follows: PTPN6 (BC002523) forward-TGGCGTGGCAGGAGAACAG and reverse-GCAGTTGGTCACAGAGTAGGGC; GADPH (NM002046) forward-ACCACCATGGAGAAGGC and reverse-GGCATGGACTGTGGTCATGA; COX2 (NM000963) forward-CTGGCGCTCAGCCATACAG and reverse-CGCACTTATACTGGTCAAATCCC; iNOS (NM000625) forward-TTCAGTATCACAAGTTAAAATCC. Similar amplification procedures and data computation were followed as described above. No PCR products were generated from genomic *versus* cDNA template. Melting curve analysis was performed to confirm the purity of the PCR products. The relative expression of iNOS, COX2 and PTPN6 was normalized to GAPDH using the ΔCT method [relative expression = 2^−ΔCT^, where ΔCT = CT(iNOS, COX2, PTPN6) − CT(GAPDH)]. Predicted cycle threshold values were exported directly into Excel worksheets for analysis. Relative changes in gene expression were determined by the 2^−ΔΔCT^ method as described previously and reported as the (n-fold) difference relative to the value for a calibrator cDNA (control) prepared in parallel with the experimental cDNAs. Data are representative of three different experiments each run in triplicate and are presented as the mean ± SEM of triplicates. DNA was denatured at 95°C for 2 min. followed by 40 cycles of 30 sec. at 95°C together with 30 sec. at 60°C. The experiments were repeated twice with consistent results.

### Western blot analysis

Total protein extracts were prepared by treating cells with lysis buffer (RIPA). Nuclear extracts were prepared as previously described 23. Proteins were quantified using the Bradford method. Western blot analysis was performed as described previously 24 using the following primary antibodies: anti-pSHP1, pTAK-1, pJNK, AP-1, iNOS and COX2 (Santa Cruz Biotechnology, Santa Cruz, CA, USA). Nitrocellulose was then washed in TBS, and incubated with secondary HRP-conjugated antibody (dilution 1:10,000; Pierce) for 1 hr, washed again and developed. The nitrocellulose was scanned using a computerized densitometric system (Bio-Rad Gel Doc 1000, Milan, Italy) Protein levels were normalized to the housekeeping proteins actin and tubulin (Sigma-Aldrich) to adjust for variability in protein loading, and expressed as a percentage of vehicle control.

### Electrophoretic mobility shift assay

Electrophoretic mobility shift assay (EMSA) was used to determine the DNA binding activity of AP-1 by assaying the extent of binding of nuclear extracts to AP-1 consensus sequence (5′-CGC TTG ATG ACT CA G CCG GAA-3′). FAM labelled and not labelled oligonucleotides were purchased from IDT. Briefly, U937 cell membrane were disrupted with lysis buffer (10 mM Tris-HCl pH 8.0, 60 mM KCl, 1 mM EDTA, 1 mM, dithiothreitol, 100 μM PMSF, 0.1% NP-40). After centrifugation at 2500 r.p.m. for 4 min., nuclei were washed with lysis buffer without NP-40. Subsequently, nuclear proteins were extracted with nuclear extraction buffer (20 mM Tris-HCl pH 8.0, 420 mM NaCl, 1.5 mM MgCl_2_, 0.2 mM EDTA, 25% glycerol). After centrifugation, protein concentration was estimated with Bradford assay. Nuclear proteins (4 μg) were incubated with 0.001 U/ml of poly-dIdC, in binding buffer (10 mM Tris-HCl, pH 7.5, 1 mM EDTA, 0.1 mM DTT, 10% glycerol), for 10 min. at RT. Then each sample was splitted in two: in one was added 5′-6FAM labelled AP-1 probe (100 nM) and incubated for 25 min. at RT, while in the other was added not labelled AP-1 probe (10 μM), before the addition of the labelled probe. Samples were loaded on 5% polyacrylamide TBE gel and run at 150 V. Immages were acquired on UVItec gel analysis system and data were analysed with Alliance 4.7 software.

### Measurement of PGE2 Release

U937 cells were cultured at ∽80% density in 12-well plates with or without LPS (10 μg/ml) in RPMI medium containing 10% FBS for 24 hrs. The medium was then changed and collected at the time intervals indicated for measuring PGE_2_ using an enzyme immunoassay (Arbor Assays, Ann Arbor, MI, USA) according to the manufacturer’s instructions. Briefly, control and samples were added to each well and incubated for 15 min. at room temperature and overnight at 4°C with primary antibody and conjugate. After washing, substrate solution was added to each well for 30 min. at room temperature. Finally, ‘stop solution’ was added, and the optical density of each well was determined within 30 min. using a microplate reader (wavelength 450 nm) The standards used were in the range of 12.5–400 pg/ml (detection limit of 16.8 pg/ml) for PGE_2_ (sensitivity of 10.9 pg/ml).

### NOS activity

Nitric oxide formation from NOS was monitored by the oxyhemoglobin assay as described previously 25. A typical assay mixture for NOS contained 10 μM l-arginine, 1 mM MgCl_2_, 100 μM NADPH, 6.5 μM tetrahydrobiopterin, and 3 mM oxyhemoglobin in 100 mM HEPES (pH 7.5) iNOS activity was assessed as described above, but in calcium-free conditions. All assays were in a final volume of 1 ml and were initiated with enzyme. Nitric oxide reacts with oxyhemoglobin to yield methemoglobin which is detected at 576 nm (*e* = 12,000 M^−1^ cm^−1^) on a Perkin-Elmer LamdaBIO UV–vis spectrophotometer.

### Statistical analysis

All results were expressed as means ± SD. Repeated-measure anova was performed to compare means between groups. A probability of null hypothesis of <5% (*P* < 0.05) was considered as statistically significant.

## Results

### Cytotoxicity of Verbascoside

**Figure 1:**
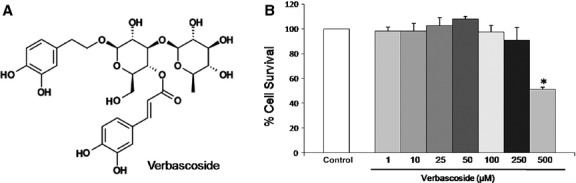
(A) Chemical structure of Verbascoside. (B) Cytotoxic effect of verbascoside tested on cells from U937 cells line. Cells were treated with Verbascoside (0, 10, 25 50, 100, 250 and 500 μM) for 24 hrs and subjected to MTT assay to analyse cell cytotoxicity. Data are presented as means ± SD for triplicate experiments. *P ≤ 0.05 *versus* control cells.

Verbascoside, a PP compound, is a purified form of *Verbascum thapsus* (Fig.1A) We first measured the cytotoxicity of verbascoside in U937 cells by using the MTT assay. Cells were treated with various concentrations of verbascoside (1, 10, 25, 50, 100, 250 and 500 μM) for 24 hrs. As shown in Figure1B, verbascoside at concentrations from 10 to 250 μM do not interfere with cell viability, while it reduced the cell viability at a concentration of 500 μM by at least 50% (*P* < 0.05) As a whole, verbascoside showed relatively low cytotoxic effects on this cell line. Data are presented as means ± SD of cell survival compared to controls (*P ≤ 0.05, *n* = 3).

### Verbascoside suppresses nuclear AP-1 expressions in an LPS-stimulated U937 cell line, *via* TAK1/JNK

**Figure 2:**
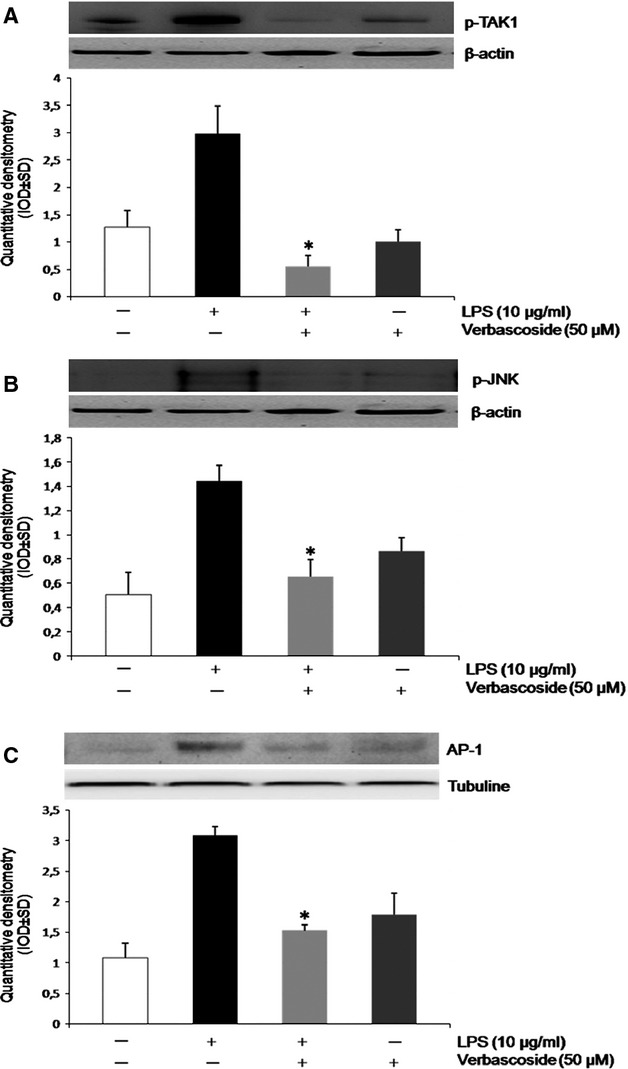
Effect of verbascoside on nuclear AP-1 expression in LPS-stimulated U937 cell lines, *via* TAK1/JNK. (A) pTAK1 protein expression in the U937 cell line using western blot analysis. Cells were treated or not treated with LPS (10 μg/ml) and/or Verbascoside (50 μM) At the top, a representative image of the Western blot experiment relative to the expression of pTAK1. At the bottom, averaged band density of pTAK-1 from U937 cells normalized *versus* ß-actin. The data represent relative density normalized to ß-actin (means ± SD, *n* = 3 each group). *P < 0.05 *versus* LPS-treated cells. (B) Representative Western blot and densitometric analysis of pJNK (U937 cells lines) ß-actin were used as internal standards. Data are means ± SD of three determinations for each experiment (*n* = 3). *P < 0.05 *versus* LPS treated cells. (C) Western-blot analysis of nuclear protein extracts. Representative image of immunoblots showing the expression of AP-1 (top) after LPS treatment 10 μg/ml or LPS + Verbascoside (50 μM) and verbascoside treatment. The histogram (bottom) represents relative density normalized to tubuline (mean ± SD, *n* = 3 each group). *P < 0.05 *versus* LPS treated cells.

Because TAK1 has been implicated in the regulation of MAPK phosphorylation by LPS treatment, we investigated whether verbascoside could suppress the LPS-induced phosphorylation of TAK1 in U937 cells. The activation of TAK-1 and JNK MAPKs was detected by Western blotting assay using the phosphorylation level as an index of enzyme activity. As shown in Figures2A and B, treatment of U937 with verbascoside (50 μM) markedly prevented the LPS-induced increase in TAK-1 and JNK phosphorylation. Together, these results suggest that regulation of TAK1 and JNK MAPK signalling cascade is a possible mechanism underlying the effect of verbascoside in LPS-activated U937. Band densities of p-TAK1 and p-JNK are expressed as relative to the control (β-actin). To evaluate the effect of verbascoside on AP-1 expression, we performed a western blot analysis on nuclear extract proteins. As illustrated in Figure2C, AP-1 nuclear translocation was induced at significantly higher levels by LPS treatment rather than in control cells. As expected, verbascoside co-treatment significantly inhibits AP-1 activation, as suggested by the reduction in nuclear translocation. The data are means ± SD (*n* = 3); *P < 0.05 *versus* LPS treated cells.

### Verbascoside increases SHP-1 Phosphorylation

**Figure 3:**
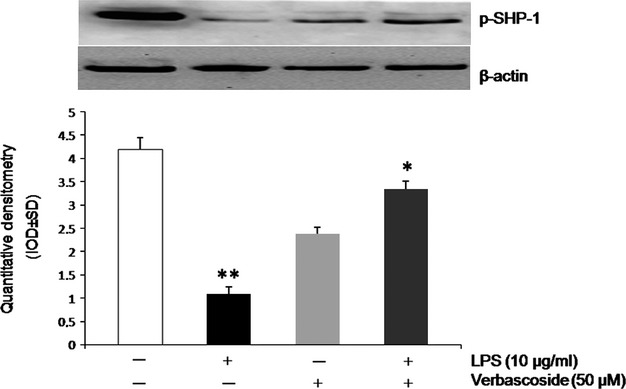
Verbascoside induces p-SHP-1 expression in U937 cells. At the top, there is a representative image of western blotting experiment for p-SHP-1 from 6 gels using 3 separate pools of protein extracted from U937 cells. Averaged band density of p-SHP-1 from U937 cells normalized *versus* β-actin. Cells were treated or not treated with LPS and/or Verbascoside. *P < 0.05 *versus* LPS treated cells; **P < 0.05 *versus* control cells.

Because previous studies reported that SHP-1 tightly regulates both NF-kB and AP-1 26, we subsequently analysed the expression of SHP-1 as a possible target in the recovery of cellular damage in the activated U937 cell line. To test this hypothesis, immunoblotting analysis was performed. Cells were incubated with Verbascoside 50 μM, and whole-cell extracts were prepared and examined for the phosphorylated form of SHP-1 by Western blot analysis. We used an antibody against pSHP-1^Tyr536^. As shown in Figure3, Verbascoside significantly induced SHP-1 activation in U937 cells, while the level of pSHP-1^Tyr536^ was reduced by LPS.

### Verbascoside-induced Inhibition of TAK-1 Activation is Reversed by Gene Silencing of SHP-1

**Figure 4:**
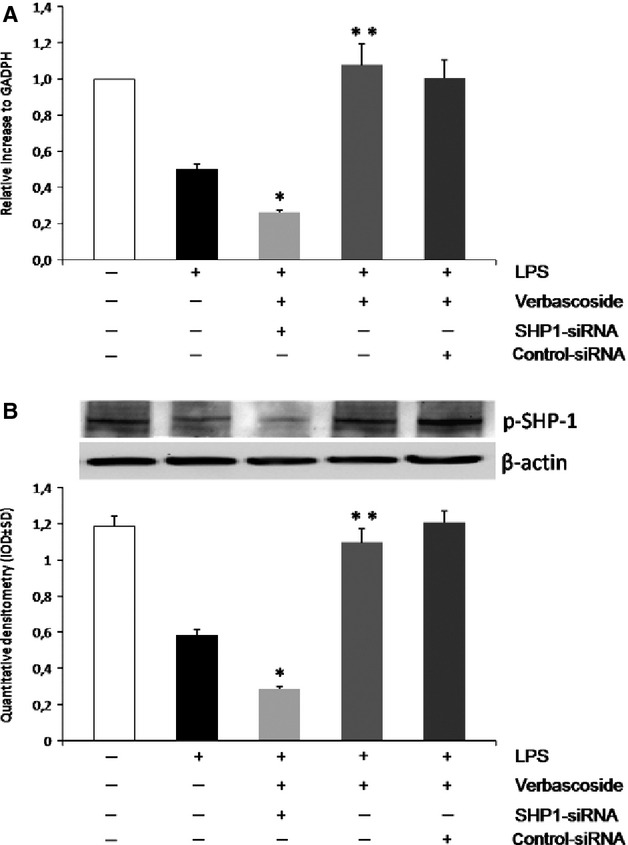
Effects of SHP-1 silencing on the ability of verbascoside to induce the p-SHP-1 expression in U937 cells. The effectiveness of SHP-1 siRNA in lowering the SHP-1 expression was evaluated by both real-time RT-PCR and western blotting. (A) At the top, real-time RT-PCR quantification of *PTPN6* mRNA 24 hrs after the silencing procedure. Values are means ± SD of three experiments. RT-PCR analysis with SHP1 and GADPH primers; (B) At the bottom, representative western blot and densitometric analysis of p-SHP-1 protein 48 hrs after the silencing procedure. Values are means ± SD of three experiments. Western blotting analysis with anti-SHP1 and anti-β-actin antibodies.

**Figure 5:**
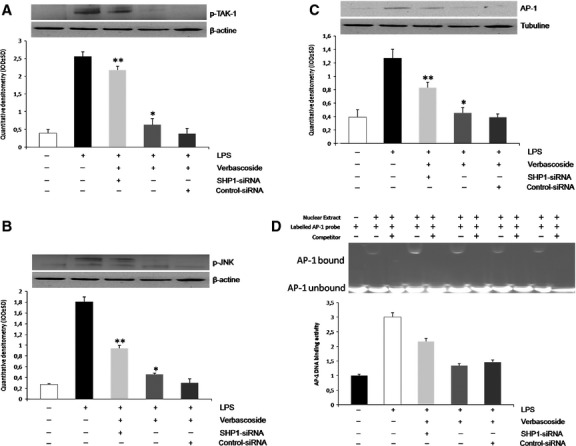
Verbascoside-induced Inhibition of TAK-1 activation is reversed by Gene Silencing of SHP-1. (A) Representative western blotting shows how verbascoside-blocked TAK-1 phosphorylation is inhibited by SHP1-siRNA. The relative expression of pTAK-1 is compared with β-actin under each experimental condition. At the bottom, densitometric analysis of the effect of SHP-1 siRNA treatment on TAK-1 protein expression in untransfected cells (control cells), in LPS-treated cells, and in siSHP-1 cells treated with LPS+ Verbascoside. *P < 0.05 *versus* LPS. **P < 0.05 *versus* control-siRNA. (B) The representative western blot analysis shows that TAK1-induced phosphorylation of JNK is affected by SHP1-siRNAs. Actin shows equal loading protein. Densitometric quantification of Western blots is given as mean values ± SD (*n* = 3) *P < 0.05 *versus* LPS. **P < 0.05 *versus* control-siRNA. (C) Western blot analysis was performed on nuclear extract of U937 cells using antibodies against AP-1 and tubuline after transfection with SHP-1 or control-siRNA. Values are means ± SD of three experiments. *P < 0.05 *versus* LPS. **P < 0.05 *versus* control-siRNA. (D) EMSA analysis was used to test the AP-1 DNA binding activity. Gel shift assay, performed on nuclear extract (4 μg) of U937 from control, verbascoside-treated and SHP1-siRNAs cells, showed down-regulation of AP-1 activity in cells treated with verbascoside. Values are means ± SD of three experiments. *P < 0.05 *versus* LPS. **P < 0.05 *versus* control-siRNA.

To confirm that Verbascoside down-regulates LPS-induced TAK-1, JNK and AP-1 activation *via* the activation of SHP-1, the effect of SHP-1 specific siRNA on LPS-induced TAK-1, JNK and AP-1 expression was examined. Firstly, we confirmed the effectiveness of silencing by the inhibition of SHP-1. The U937 cell line was transiently transfected with siRNAs against SHP-1 (siSHP-1), or with a non-targeting control siRNA (cont-siRNA), and then treated with 10 μg/ml of LPS or co-treated with LPS and verbascoside (Fig.4) Transfection of SHP-1-specific siRNA clearly depleted both for gene and SHP-1 protein expression, respectively, 24 hrs after transfection (for mRNA analysis) and 48 hrs (for protein synthesis; Fig.4). In particular, SHP-1 gene silencing resulted in the inactivation of TAK-1 and JNK, as indicated by the dephosphorylation of p-TAK1 at Ser 192 and p-JNK at Thr 183 (Fig.5A and B) In addition, the reduction in TAK1/JNK signalling expression slightly reduced the AP-1 activation (Fig.5C). To confirm the effective nuclear translocation and activation of these transcription factor we performed an EMSA (Fig.5D). These data indicate that the effect of Verbascoside on TAK-1/JNK/AP-1 expression is associated with the activation of SHP-1 involved in the regulation of the LPS response.

### Gene silencing of SHP-1 regulated iNOS and COX2 activity and expression

**Figure 6:**
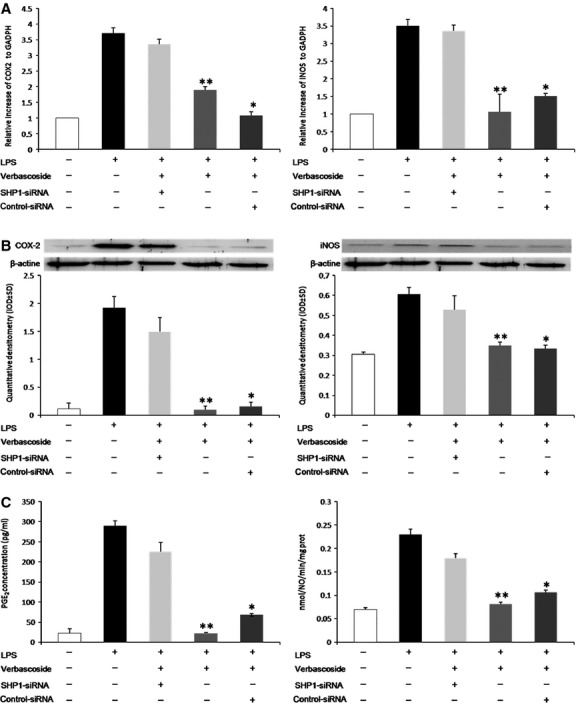
The effect of SHP-1 silencing on COX-2 and iNOS expression and activity in U937 cells. (A) The effects of verbascoside on LPS-induced COX-2 and iNOS mRNA expressions in U937 cells. Cells were pre-treated with verbascoside (1 hr) and then treated with LPS (10 μg/ml) Total RNA was isolated and iNOS and COX-2 mRNA were determined by a q-PCR. GAPDH expression used as reference gene. The results were expressed as mean ± SD of three independent experiments. *P < 0.05 *versus* LPS; **P < 0.05 *versus* control-siRNA. (B) Effects of verbascoside on LPS-induced iNOS and COX-2 protein levels in U937cells. Cells were pre-treated with verbascoside for 1 hr. U937 cells were then incubated with LPS (10 μg/ml, 24 hrs) Protein extracts were performed and subjected to Western blot using anti-iNOS and COX-2 antibodies. β-actin was used as the internal control. *P < 0.05 *versus* LPS; **P < 0.05 *versus* control-siRNA. (C) Effects of verbascoside on nitric oxide and PGE_2_ levels in U937 cells stimulated with LPS. Cells treated with LPS showed a significant increase in nitric oxide and PGE_2_ levels, while co-treatment with verbascoside prevented this increase (anova; *P < 0.05 *versus* LPS; **P < 0.05 *versus* control-siRNA.

To confirm the effect of verbascoside on reducing the development of inflammation through the activation of SHP-1, we assessed the activity and expression of inducible proteins such as iNOS and COX-2. To assess the effect of verbascoside on iNOS and COX-2 mRNA expression, q-PCR experiments were performed. Interestingly, the U937 cells pre-treated with verbascoside showed significant downsizing of the LPS-mediated induction of iNOS and COX-2 (Fig.6A), while verbascoside did not affect the expression of the housekeeping gene GAPDH. We then investigated whether the inhibitory effect could be extended to the expression of iNOS and COX-2 proteins. In unstimulated U937 cells, iNOS and COX-2 protein levels were undetectable. However, in response to LPS, their expression was markedly up-regulated. Furthermore, verbascoside significantly inhibited LPS-stimulated iNOS and COX-2 up-regulation (Fig.6B; *P < 0.001 *versus* control; #*P* < 0.001 *versus* LPS alone) Nitric oxide formation from NOS was monitored by the oxyhemoglobin assay. While LPS alone markedly increased nitric oxide production compared to control cells alone, the compound significantly reduced the level of nitric oxide production in LPS-induced U937 cells at a concentration of 50 μM (Fig.6C) COX-2 activity was measured by evaluating the PGE2 concentration. Verbascoside (50 μM) inhibited the PGE2 synthesis in the activated U937 cell line (Fig.6C) Analysis of NOS2 and COX2 activity confirmed that verbascoside-induced inactivation of NOS2 and COX2 activity can be mediated by SHP-1. Finally, SHP-1 depletion resulted in a significantly higher induced release of nitric oxide and PGE2 in the medium of cultured cells. In cultured monocytes, LPS significantly increased the activity of COX-2 and NOS-2, but the pre-transfection of siSHP-1 blocked the effect of Verbascoside on COX-2 and NOS-2 activity.

## Discussion

Medicinal plants are traditionally used in folk medicine as natural healing remedies with therapeutic effects such as the prevention of cardiovascular diseases and inflammation disorders, or reducing the risk of cancer 27. There is growing evidence that PPGs, like other plant polyphenols in general and PPs in particular, are powerful antioxidants whether by direct scavenging of reactive oxygen and nitrogen species, or by acting as chain-breaking peroxyl radical scavengers 28,29. Verbascoside is a natural compound present in plants used in traditional medicine. The biological properties of verbascoside have been described in literature and comprise of a wide range of activities, including antioxidant, anti-inflammatory, photoprotective and chelating actions 30. The protein tyrosine phosphathase SHP-1 is a crucial negative regulator of cytokine signalling and inflammatory gene expression in both the immune system and the central nervous system. Pesce *et al*. 16 have recently demonstrated that SHP-1 expression was deficient in PBMCs from schizophrenia patients (SC) and that the promoter region analysed, displayed significant hypermethylation. Moreover, *ex vivo* PBMCs from SCs showed higher IKK/NF-kB the signalling of healthy individuals, and significant induction of IL-1β, IL-2 and TNF-α in a conditioned medium of *ex vivo* PBMCs, reflecting the observation of increased nuclear translocation of p-p65 subunits of NF-kB. The functions of SHP-1 and SHP-2 during cell injury and inflammation are not easily defined, since these PTPs appear to possess dual roles, either enhancing or inhibiting cell survival under a variety of environmental conditions. In particular, SHP-1 has been shown to influence the inflammatory cell function by promoting the survival of macrophages. We previously found that, in THP-1 cells stimulated with pro-inflammatory cytokines, treatment with verbascoside decreased iNOS activity by reducing NF-kB activation and nuclear translocation, preventing ROS related to damage 31. Although NF-κB is considered to be the main transcription factor controlling the activation of LPS-responsive proteins, the transcription factor AP-1 is also considered an important regulator of the inflammatory response 32. Several findings suggest that AP-1 activation could occur *via* up-stream induction of the TGF-β activating kinase 1 (TAK1) Because TAK1 is able to phosphorylate both upstream MAPK and IKK, this kinase is considered to be at the branching point of the two pathways. In parallel, active TAK1 phosphorylates MAPK kinases MKK3/6 and MKK4/7, which subsequently phosphorylate JNK MAPKs. Activated JNK kinases induce gene transcription by phosphorylation of nuclear transcription factors such as AP-1 33,34. In order to understand better the signalling mechanism underlying the effect of verbascoside, the modulation of TAK1, JNK and AP-1 expression was monitored. Our results show that verbascoside treatment prevents the phosphorylation of tak1 caused by LPS and inactivates it. This leads to a significant decrease in the protein level of pJNK and consequently to the inactivation of the transcriptional factor AP-1, as shown in Figure2. Studies using various experimental systems have highlighted that SHP-1 is an important negative regulator of several kinases, such as Erk1/Erk2 MAPKs 35, p38 MAPK 36 and JNK 37, and that these kinases play a role in IFN-γ- and/or LPS-induced nitric oxide generation 38–42. Because previous studies reported that SHP-1 regulates AP-1 26, we subsequently analysed the expression of SHP-1 as a possible target in the recovery of inflammatory damage in an activated U937 cell line. As shown in Figure3, stimulation with LPS dephosphorylates and inactivates SHP-1, while verbascoside treatment restores the kinase activity which is fundamental for the resumption of cell function in response to inflammatory damage. This is probably responsible for the reduction in phosphorylation of the JNK MAPKs TAK-1 system and consequent inhibition of the AP-1 expression. To demonstrate our hypothesis, we silenced the gene that codes the PTPN6 SHP-1 protein, demonstrating how the absence of transcription perfectly correlates with the phosphorylation of the TAK-1/JNK system, resulting in the intensification of the AP-1 expression (Fig.4). Verbascoside treatment did not reduce LPS-induced TAK-1/JNK/AP-1 protein expression in cells transfected with SHP-1 specific siRNA, suggesting that the inhibition observed with verbascoside is dependent on SHP-1. In LPS-stimulated cells, treatment with verbascoside leads to marked phosphorylation of the SHP-1 protein (Fig.4), while restoring this system results in the inhibition of pro-inflammatory TAK-1/JNK/AP-1 signalling, as illustrated in Figure5. Finally, to confirm the role of verbascoside as a molecule able to restore cells from stress mediated by an LPS-induced inflammatory state, we assessed the expression and activity of inducible genes, iNOS and COX, transcription of which is strictly mediated by AP-1. The pre-transfection of siSHP-1 blocks the effect of verbascoside on the expression and activity of COX-2, NOS-2, as shown in Figure6. In the above conclusion, these data highlight an unexpected role by SHP-1 in regulating TAK-1/JNK/AP-1 signalling, and suggests an opportunity for developing therapies, activating PTPs for the treatment of inflammatory diseases which entail modulation of the SHP-1/TAK1 system. Moreover, we report after finding, that verbascoside attenuates LPS-induced pro-inflammatory mediator production in activated U937 cells *via* SHP-1 signalling, preventing cell damage induced in activated U937.

Our results should encourage further studies on this natural compound. Further signalling pathways could be explored in which it may be involved, and this would provide additional mechanistic insight into the therapeutic potential of verbascoside in treating inflammatory diseases.
